# CorDiffViz: an R package for visualizing multi-omics differential correlation networks

**DOI:** 10.1186/s12859-021-04383-2

**Published:** 2021-10-09

**Authors:** Shiqing Yu, Mathias Drton, Daniel E. L. Promislow, Ali Shojaie

**Affiliations:** 1 Department of Statistics, University of Washington, NE Stevens Way, Seattle, WA 98195 USA; 2 Department of Mathematics, Technical University of Munich, Boltzmannstraße, 85748 Garching bei München, Germany; 3 Departments of Pathology and Biology, University of Washington, NE Pacific St, Seattle, WA 98195 USA; 4 Department of Biostatistics, University of Washington, NE Pacific St, Seattle, WA 98195 USA

**Keywords:** Correlation networks, Data integration, Differential correlations, Omics, Undirected graphs, Visualization

## Abstract

**Background:**

Differential correlation networks are increasingly used to delineate changes in interactions among biomolecules. They characterize differences between omics networks under two different conditions, and can be used to delineate mechanisms of disease initiation and progression.

**Results:**

We present a new R package, CorDiffViz, that facilitates the estimation and visualization of differential correlation networks using multiple correlation measures and inference methods. The software is implemented in R, HTML and Javascript, and is available at https://github.com/sqyu/CorDiffViz. Visualization has been tested for the Chrome and Firefox web browsers. A demo is available at https://diffcornet.github.io/CorDiffViz/demo.html.

**Conclusions:**

Our software offers considerable flexibility by allowing the user to interact with the visualization and choose from different estimation methods and visualizations. It also allows the user to easily toggle between correlation networks for samples under one condition and differential correlations between samples under two conditions. Moreover, the software facilitates integrative analysis of cross-correlation networks between two omics data sets.

## Background

Correlations between *omics* measurements are widely used to interrogate mechanisms of biological interactions. *Differential correlation networks* capture differences between omics correlations in two populations/conditions, e.g., cases and controls [1, 2]. They can thus be used to gain insight into aberrations in biological processes and mechanisms of disease initiation and progression [3]. They have also been instrumental in gaining insights into biological responses to environmental factors [4, 5] or functional consequences of mutations [6, 7]. This has led to the development of multiple methods for differential correlation analysis in recent years [8–14]; see [2, 15] for more comprehensive review. However, software tools for estimating and visualizing differential correlation networks have received less attention. Moreover, existing software either only focus on a single omics data type (commonly, mRNA expressions) and do not facilitate *integrative analysis of multiple omics data* [10, 13, 14, 16–19], or only provide static visualizations (e.g. heatmaps) [16, 17, 19–21].

Our CorDiffViz package provides a simple tool for estimation and interactive visualization of correlation networks and their differences. It also facilitates omics data integration via unifying visualizations for single and differential cross-correlation networks among two omics data types. Differential cross-correlation networks have been examined recently [11, 12, 21]; however, this work only provides visualization for a single correlation at each time [21], or has no publicly available package or visualization tools [11, 12] .

Correlations in multi-omics settings can be compared via a direct approach: concatenating the multiple omics measures into a single data set and using existing software tools to compare correlations in such concatenated data across two populations. However, by developing a tailored method for cross-omics correlation analysis, our software has at least two advantages over the direct approach. First, the direct approach requires estimating and testing many more correlations ($O(p_X + p_Y)^2$ for two data sets ${\varvec{X}}$ and ${\varvec{Y}}$ with $p_X$ and $p_Y$ variables), whereas our approach processes only $O(p_Xp_Y)$ correlations. This not only saves a significant amount of computation but also narrows the focus of multiple testing adjustments to the correlations of interest, leading to a power gain for statistical tests. Second, in addition to the interactive visualization that is currently not widely available, our software offers tailored visualization for cross-omics correlation.

**Fig. 1:**
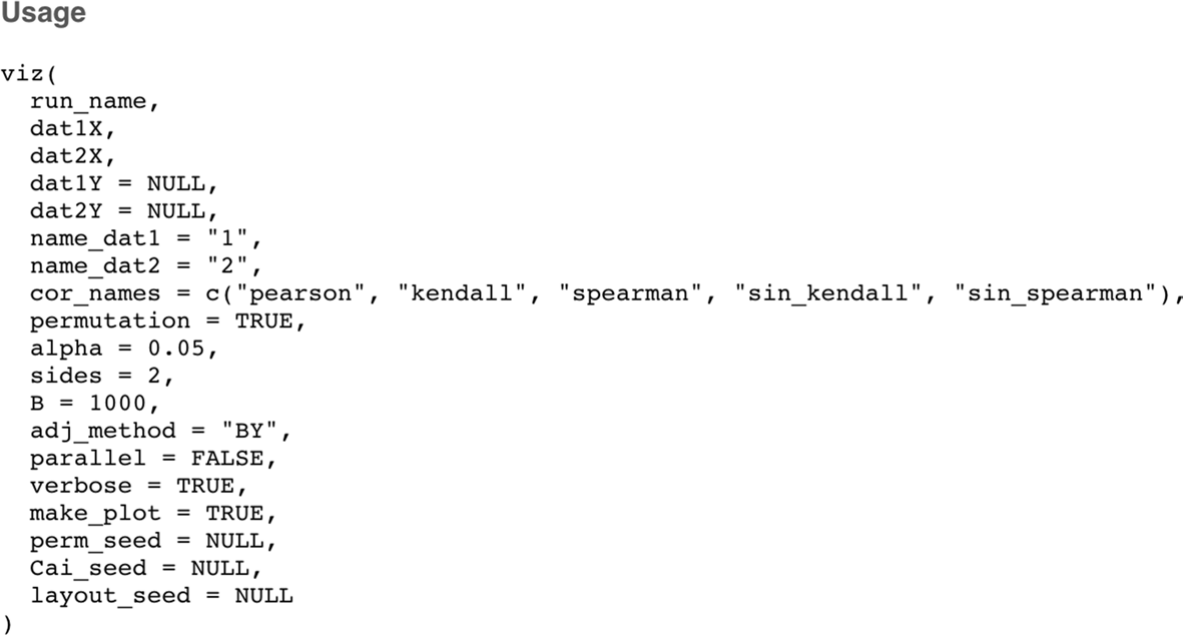
A screenshot of the function prototype of viz(), the main user-facing function in the CorDiffViz package

Another benefit of CorDiffViz compared with existing tools is that, in addition to Pearson correlation, it implements rank-based correlation measures that are better suited for non-Gaussian observations commonly encountered in omics data. The package provides both parametric and permutation tests for these correlation types. Unlike existing software tools, the resulting *p*-values, together with the implemented adjustments for multiple testing and false discovery rate (FDR) control, provide formal inference for differential correlation/cross-correlation analysis by accounting for the uncertainty in differential correlation measures. These estimation and visualization capabilities are particularly designed for sparse (differential) correlation matrices, where most (changes in) correlations are zero or negligible. The user has access to interactive visualization of both single condition and differential correlation networks by just calling one simple function in R [22]; see Fig. 1.

## Implementation

### Overview

In this section, we give an overview of the estimation methods implemented in the CorDiffViz package for differential correlation analysis. To simplify the user interface, all methods have been implemented in a single function, viz() in R. The full list of its options is displayed in Fig. 1. In what follows, we describe the estimation methods available through the function, along with their various options.

Consider two sets of omics measurements (e.g., mRNA expression and protein abundances) with data matrices ${\varvec{X}}^{(1)}\in \mathbb {R}^{n_1\times p_X}$ and ${\varvec{Y}}^{(1)}\in \mathbb {R}^{n_1\times p_Y}$ (with $n_1$ units and $p_X$ and $p_Y$ measurements, respectively) coming from one population and ${\varvec{X}}^{(2)}\in \mathbb {R}^{n_2\times p_X}$ and ${\varvec{Y}}^{(2)}\in \mathbb {R}^{n_2\times p_Y}$ from another (with $n_2$ units and $p_X$ and $p_Y$ measurements, respectively). We estimate and visualize $\mathrm {cor}\big ({\varvec{X}}^{(1)},{\varvec{Y}}^{(1)}\big )$, $\mathrm {cor}\big ({\varvec{X}}^{(2)},{\varvec{Y}}^{(2)}\big )$ and $\mathrm {cor}\big ({\varvec{X}}^{(1)},{\varvec{Y}}^{(1)}\big )-\mathrm {cor}\big ({\varvec{X}}^{(2)},{\varvec{Y}}^{(2)}\big )$, where $\mathrm {cor}\big ({\varvec{X}},{\varvec{Y}}\big )$ denotes the matrix of correlations between column vectors of ${\varvec{X}}$ and ${\varvec{Y}}$. It is worth noting that the software can also be used for analyzing one omics data type, e.g., mRNA expression levels, by simply excluding the second sets of data, ${\varvec{Y}}^{(1)}$ and ${\varvec{Y}}^{(2)}$ from the above expressions and focusing on, e.g. $\mathrm {cor}\big ({\varvec{X}}^{(1)}\big )$. The NULL default values for dat1Y and dat2Y in Fig. 1 corresponds to this simpler problem.

We consider five measures of correlation: (i) Pearson’s product-moment correlation *r*, (ii) Kendall’s $\tau$, (iii) Spearman’s $\rho$, (iv) the $\sin$-transformed $\tau$, $\sin \left( \pi \tau /2\right)$, and (v) the $\sin$-transformed $\rho$, $2\sin \left( \pi \rho /6\right)$. For continuous distributions obtained from arbitrary monotone transformations of the original data (known as Gaussian copulas), the transformed rank correlations from (iv) and (v) consistently estimate an underlying Pearson’s *r* [23, 24]. Under non-Gaussian models, these correlation types, along with the corresponding tests described below, provide more robust inference for differential correlations compared to the *z*-tests for Pearson correlations in [16].

For each correlation measure, the user can choose from the following estimates for visualization: (a) the raw (differential) correlation matrices, (b) the matrices thresholded using parametric tests, and (c) the matrices thresholded using permutation tests. For (b), the limiting distribution of each sample correlation is used for *z*-tests that are further adjusted for multiple testing; entries in the matrices that are not statistically significant are set to 0. The user can choose the adjustment method through the adj_method argument (see Fig. 1) from those supported by p.adjust() in base R. Denoting by ${\mathcal {N}}$ the standard normal distribution, and by ${\mathcal {T}}_{n-2}$ the Student’s *t*-distribution with $n-2$ degrees of freedom, the limiting null distributions for our correlation estimates are determined as follows.

(I) Pearson’s correlation: under the Fisher transformation, $\sqrt{n-3}\log ((1+r)/(1-r))/2\rightarrow _d{\mathcal {N}}$ [25, 26]
(II) Kendall’s $$\tau$$: $$\sqrt{9n(n-1)/(2(2n+5))}\tau \rightarrow _d {\mathcal {N}}$$ [26];
(III) Spearman’s $$\rho$$: $$\sqrt{n-2}\rho /\sqrt{1-\rho ^2}\rightarrow _d{\mathcal {T}}_{n-2}$$ [26];
(IV) $\tau '=\sin (\pi \tau /2)$: $\sqrt{18n(n-1)/(2n+5)}\tau '/\pi \rightarrow _d {\mathcal {N}}$ (from (II) with the delta method [27]);
(V) $\rho '=2\sin (\pi \rho /6)$: $3\sqrt{n-2}\rho '/\pi \rightarrow _d{\mathcal {N}}$ (from (III) with the delta method [27]).

For the differential correlations, we use limiting normal distributions that follow from (I)–(V) above, using the fact that $\mathrm {var}(U+V)=\mathrm {var}(U)+\mathrm {var}(V)$ for independent random variables *U* and *V*.

For (c), we use permutation tests in which samples are randomly shuffled; the user can choose the number of permutations through argument B (see Fig. 1). A random number seed for this procedure can be specified by the argument perm_seed to ensure reproducibility.

For either case, parametric or permutation tests, one can also choose to perform one-sided ($\ge 0$ or $\le 0$) or two-sided tests using the sides argument (Fig. 1). The choice between parametric and permutation tests is up to the user—we note that, permutation tests tend to be more robust to violated assumptions, while parametric tests provide higher statistical power when their assumptions are met.

Finally, for Pearson’s correlation, the user can also choose an inference procedure for high-dimensional *differential* correlation networks adapted from the method proposed by [3]. Specifically, suppose we have samples ${\varvec{X}}_j^{(t)}=\big (X_{j1}^{(t)},\ldots ,X_{jn_t}^{(t)}\big )$ and ${\varvec{Y}}_k^{(t)}=\big (Y_{k1}^{(t)},\ldots , Y_{kn_t}^{(t)}\big )$, corresponding to two omics data types (e.g., mRNA expression and protein abundances), respectively, for population $t=1,2$ and variables $j=1,\ldots ,p_X$, $k=1,\ldots ,p_Y$. For random vectors ${\varvec{V}}=(V_1,\ldots ,V_{n})$ and ${\varvec{W}}=(W_1,\ldots ,W_{n})$, let ${\hat{\sigma }}({\varvec{V}},{\varvec{W}})\equiv \frac{1}{n}\sum _{i=1}^n\left( V_i-\overline{{\varvec{V}}}\right) \left( W_i-\overline{{\varvec{W}}}\right)$ be the unadjusted sample covariance. Then the raw correlation between ${\varvec{X}}_j^{(t)}$ and ${\varvec{Y}}_k^{(t)}$ is ${\hat{r}}_{jk}^{(t)}\equiv {\hat{\sigma }}({\varvec{X}}_j^{(t)},{\varvec{Y}}_k^{(t)})/\sqrt{{\hat{\sigma }}({\varvec{X}}_j^{(t)},{\varvec{X}}_j^{(t)}){\hat{\sigma }}({\varvec{Y}}_k^{(t)},{\varvec{Y}}_k^{(t)})}$.

1 $$\begin{aligned} {\text {Define}} \quad {\hat{\xi }}({\varvec{V}},{\varvec{W}})&\equiv \frac{1}{|{\varvec{V}}|}\sum _{i=1}^{|{\varvec{V}}|}\frac{\left( \left( V_i-\overline{{\varvec{V}}}\right) \left( W_i-\overline{{\varvec{W}}}\right) -{\hat{\sigma }}\left( {\varvec{V}},{\varvec{W}}\right) \right) ^2}{{\hat{\sigma }}\left( {\varvec{V}},{\varvec{V}}\right) {\hat{\sigma }}\left( {\varvec{W}},{\varvec{W}}\right) },\nonumber \\ \lambda _{jk}^{(t)}&\equiv \tau \sqrt{\frac{\log (p_X+p_Y)}{n_t}}\left( \sqrt{{\hat{\xi }}\left( {\varvec{X}}_j^{(t)}, {\varvec{Y}}_k^{(t)}\right) }\right. \nonumber \\&\quad + \left. \frac{|{\hat{r}}_{jk}^{(t)}|}{2}\left( \sqrt{{\hat{\xi }}\left( {\varvec{X}}_j^{(t)}, {\varvec{X}}_j^{(t)}\right) }+\sqrt{{\hat{\xi }}\left( {\varvec{Y}}_k^{(t)}, {\varvec{Y}}_k^{(t)}\right) }\right) \right) ,\nonumber \\ s_{\lambda }(x)&\equiv x\left( 1-|\lambda /x|^4\right) \mathbbm {1}_{|x|>\lambda }. \end{aligned}$$

Then the thresholded differential correlation between ${\varvec{X}}_j$ and ${\varvec{Y}}_k$ for populations $t=1,2$ is defined as $s_{\lambda _{jk}^{(1)}+\lambda _{jk}^{(2)}}\left( {\hat{r}}_{jk}^{(1)}-{\hat{r}}_{jk}^{(2)}\right)$. The parameter $\tau$ in (1) is empirically chosen through cross-validation [3]. Since cross-validation involves random sampling, a corresponding seed can be specified through the argument Cai_seed (Fig. 1).

### Procedure

When calling the main function viz() (Fig. 1) in R, the package automatically estimates the (differential) correlation matrices, and performs permutation and parametric tests as instructed by the user. The user may run the function multiple times (with different arguments) on multiple datasets by assigning a different name to each run; each run can be visualized by selecting it from a dropdown menu in viz.html, which is automatically generated by the package.

The function outputs all raw data matrices, raw (differential) correlation matrices, and matrices that contain entry-wise *p*-values for the tests (with adjustments for multiple testing as selected by the user). These files store data using Javascript code and are for internal use by the HTML and Javascript files only. The user can then open viz.html to access the visualization.

In addition, the function also outputs static heatmaps as well as static plots of the (differential) undirected graphs. The randomization in the choice of layout for the latter can be controlled by layout_seed (Fig. 1).

### Visualization design

**Fig. 2:**
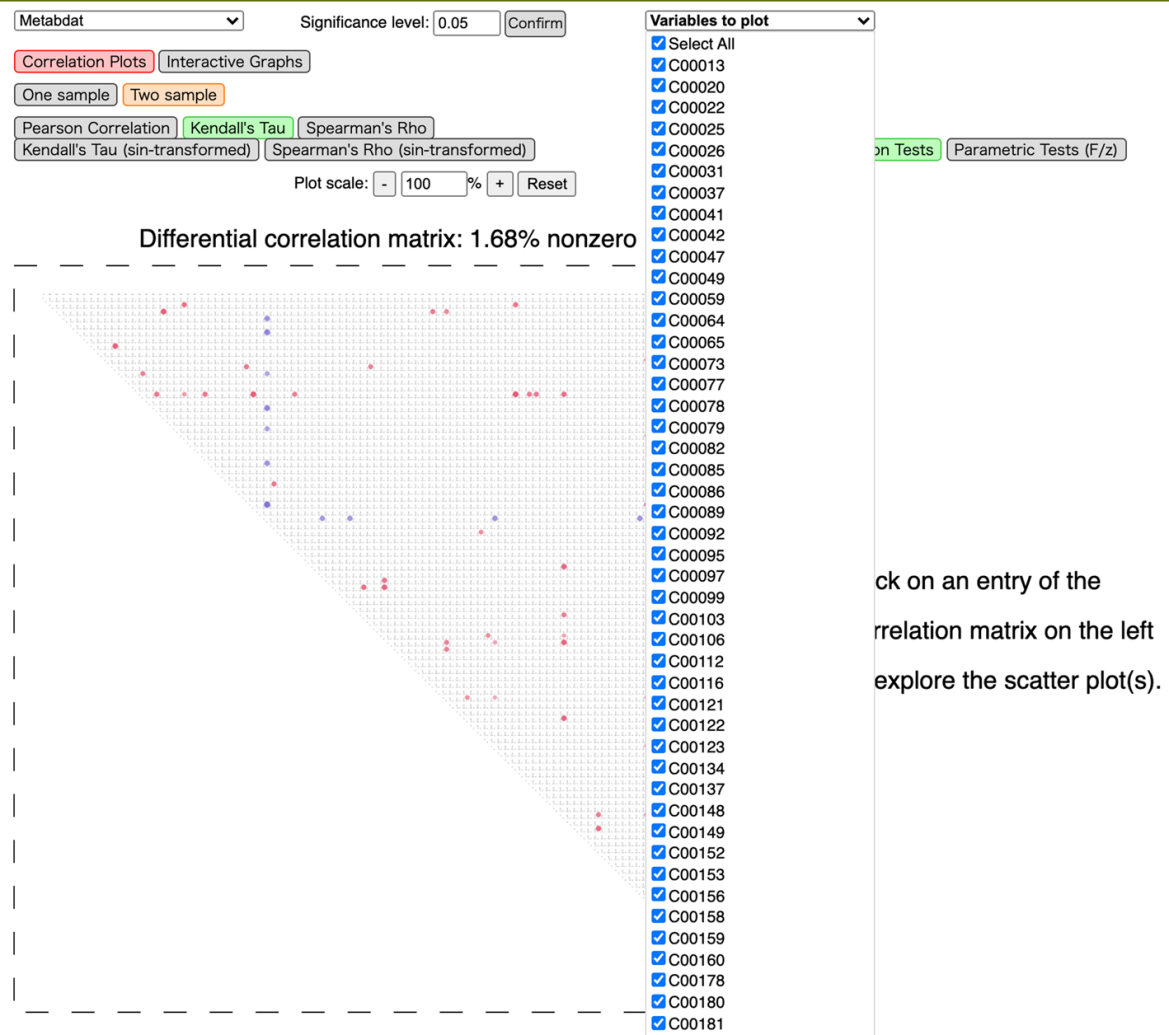
A screenshot of the dropdown menu for selecting variables to include in the correlation plots

Interactive visualization is available through the automatically copied HTML file in the current working directory in Google Chrome or Mozilla Firefox browsers. The user first needs to select the dataset (the name of the run they wish to visualize) under the current directory from a dropdown menu. Two visualization modes are currently available: *Correlation Plots* and *Interactive Networks*. In both modes, one can toggle between correlation matrices/networks for either population or the differential correlation matrix/network by enabling the “One sample” and “Two sample” buttons, respectively; for the former, the user can choose which population to visualize. One can also choose from the five correlation measures discussed above. Instead of the default 5% significance level, the user can manually enter a desired level (before adjustments for multiple testing) for the tests. In addition, under both modes, a dropdown menu allows the user to choose which variables to include (as shown in Fig. 2). The red/blue color represents a negative/positive (differential) correlation, whose magnitude is indicated by the color saturation.

## Results and discussion

In this section we demonstrate CorDiffViz in two applications. In the first application, we perform differential correlation analysis in a single omics data set, the setting that has also been considered in some of the existing software, and compare the capabilities of CorDiffViz with the existing software. The second application demonstrates how CorDiffViz can be used for differential cross-correlation analysis among two omics data sets, a setting of increasing interest for which public estimation and visualization software tools are lacking. We end this section with additional comments about the broader applicability of the package.

### Differential correlation analysis of single omics data

**Fig. 3:**
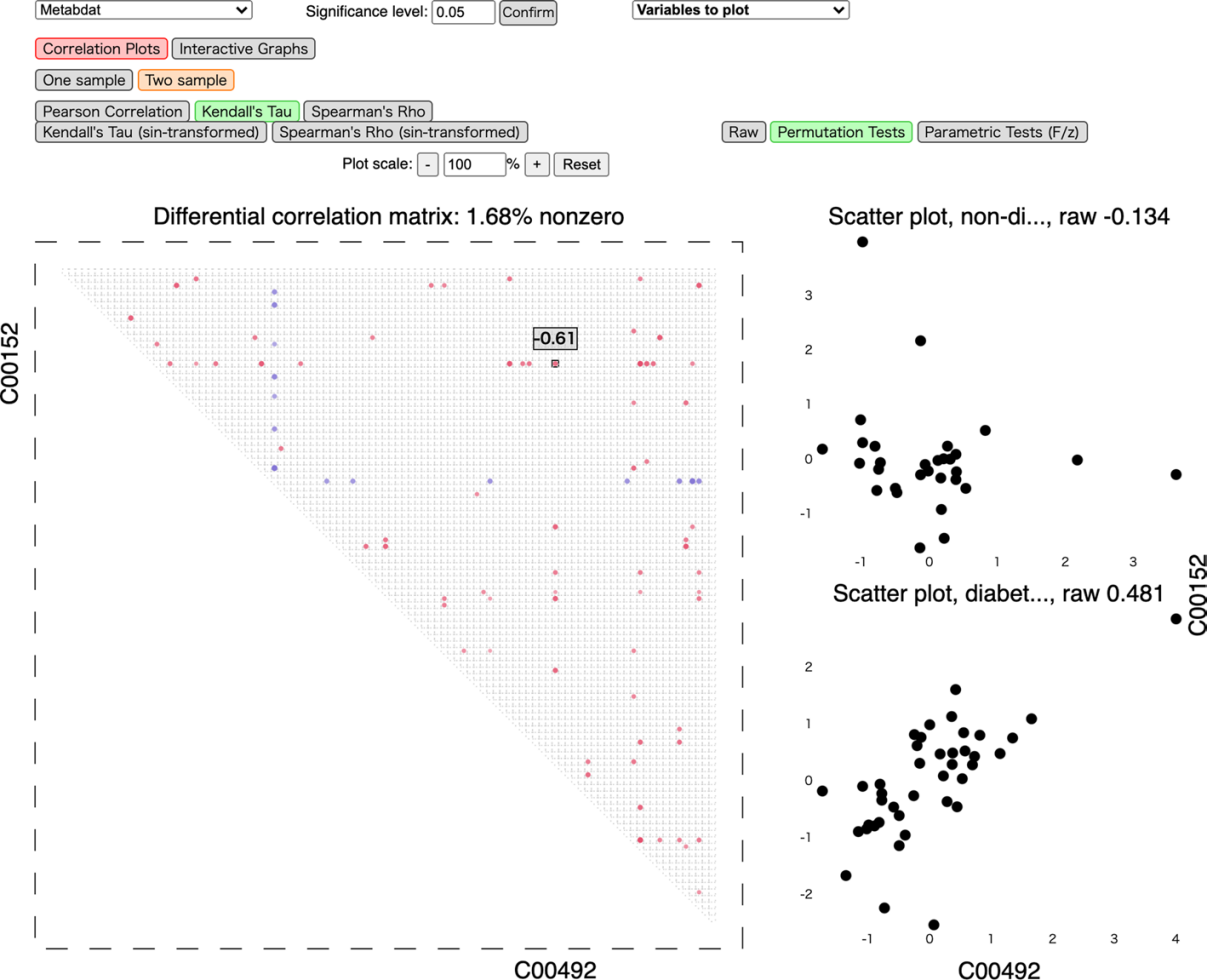
A screenshot of the interactive correlation plots using D3.js for the metabolomics dataset. Red/blue colors indicate negative/positive (differential) correlations, while color saturation and size of the circles suggest their magnitude

**Fig. 4:**
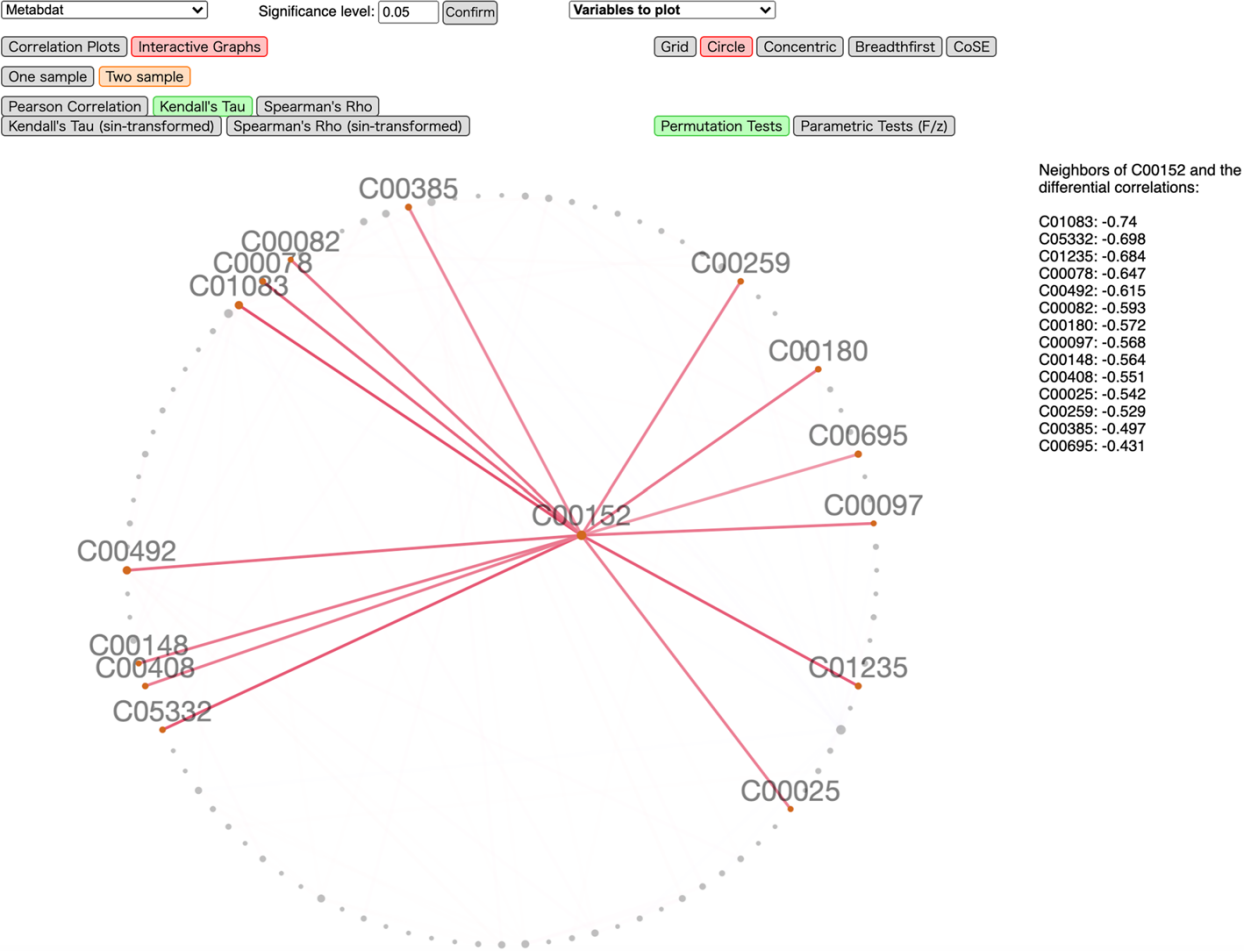
A screenshot of the interactive network plots using Cytoscape.js for the metabolomics dataset. Here *C00152* (L-Asparagine) is clicked on, with all its neighbors and edges highlighted, and all other edges hidden. Red/blue colors of the edges indicate negative/positive (differential) correlations, while color saturation suggests their magnitude

We illustrate our tool using a metabolomics dataset for mice with 100 metabolites from [28]. The data contains the metabolic profiles of 41 non-diabetic and 30 diabetic mice and has been recently analyzed in [29]. The names of the metabolites are compound IDs in the Kyoto Encyclopedia of Genes and Genomes (KEGG); for example, the hub node C00152 in Fig. 4 corresponds to L-Asparagine. In Figs. 3 and 4, we show screenshots of differential correlations using Kendall’s $\tau$ with permutation tests ($B=1000$) and *p*-values adjusted using the FDR controlling procedure of [30]. The significance level is set to 0.05.

The first visualization mode, Correlation Plots, implemented using D3.js and illustrated in Fig. 3, is a direct presentation of the raw or thresholded (differential) correlation matrices. The square/rectangle represents the matrix, with the entry in the *j*-th row and *k*-th column representing $\mathrm {cor}\big ({\varvec{X}}_{j}^{(t)},{\varvec{X}}_{k}^{(t)}\big )$ for one population $t=1,2$, or their difference. As in the figures, the user can view the value of a specific cell in the matrix, its corresponding variable names, and the corresponding scatter plots (for one population or both depending on the selection).

The second visualization mode, Interactive Networks, is implemented using Cytoscape.js and is illustrated in Fig. 4. Each node in the undirected correlation network represents a variable (feature), and an edge is present if the corresponding entry in the (differential) correlation matrix is statistically significant. Multiple network layouts are available. Each node is draggable with size positively related to the number of variables connected to it. The user can easily highlight an edge and hide all other edges, and read the (differential) correlation value and the two variables associated with it. It is further possible to highlight one node and all edges linked to it as well as the corresponding (differential) correlations, sorted in descending magnitude, as shown in Fig. 4.

**Fig. 5:**
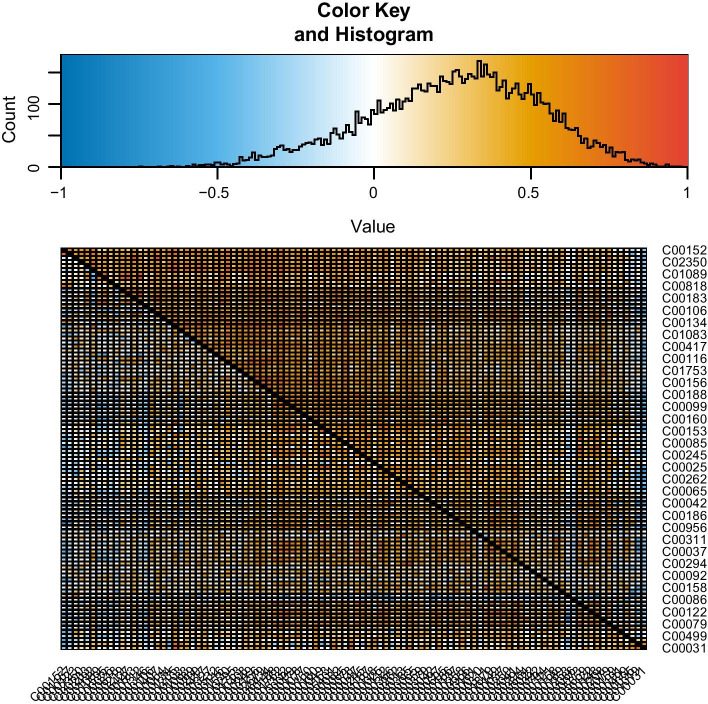
Heatmap by DGCA for the metabolomics dataset

The differential correlation matrix heatmap from DGCA [19] in Fig. 5 serves a somewhat similar purpose as the correlation plots from our tool in Fig. 3. For consistency, this heatmap is also obtained using 1000 permutations. However, only static heatmaps are supported by DGCA. Moreover, in larger data sets, even with the 100 metabolites in our dataset, the heatmap can become more difficult to discern, as there are no easy options for selecting a subset of variables to visualize. Other existing tools have similar limitations—they either provide static visualization, or visualization for pairwise correlations only.

### Differential cross-correlation analysis of two omics data sets

We now illustrate how our tool can be used to analyze and visualize differential cross-correlation networks by applying it to joint protein abundances and expression levels (based on RNAseq) from the Cancer Genome Atlas (TCGA). We denote the protein abundances as ${\varvec{X}}$ variables and the RNAseq data as ${\varvec{Y}}$ variables. We do not compare our results to DGCA in this dataset, as that package does not support multi-omics data analysis.

For simplicity, we work with the subset of samples with no missing values for all variables, as well as prostate-specific antigen (PSA) levels available. This leaves 156 samples with 127 ${\varvec{X}}$ and 4749 ${\varvec{Y}}$ variables. Since some expression levels have extreme variance while the others have many zeros, following [17], we pick the ${\varvec{Y}}$ variables that have a coefficient of variation between 0.5 and 10, after which 2679 ${\varvec{Y}}$ variables are left. We then $\log$ transform the ${\varvec{Y}}$ variables using $\log (1+y)$.

PSA is an established marker for prostate cancer. While PSA is a continuous measure, it is often dichotomized by practitioners in order to assess the risk of developing prostate cancer. Following this strategy, we split the 156 samples into 105 individuals with PSA levels $\le$ 10 nanograms per milliliter—a common threshold used for identifying those with highest risk of prostate cancer—and the remaining individuals 51with PSA levels > 10 who are at higher risk of prostate cancer.

To reduce the number of variables for better illustration, we focus on proteins and expression levels that are more clearly associated with prostate cancer. To this end, we use Wilcoxon signed-rank test as a screening method, where for each variable we calculate the *p*-value associated with the hypothesis test that the two samples have equal mean. As a simple illustration, we pick $p_X = p_Y = 40$ variables that have the highest *p*-values and no 0’s from both ${\varvec{X}}$ and ${\varvec{Y}}$. We visualize the results using Spearman’s $\rho$ with *p*-values adjusted to control the FDR [31] at significance level 0.2.

**Fig. 6:**
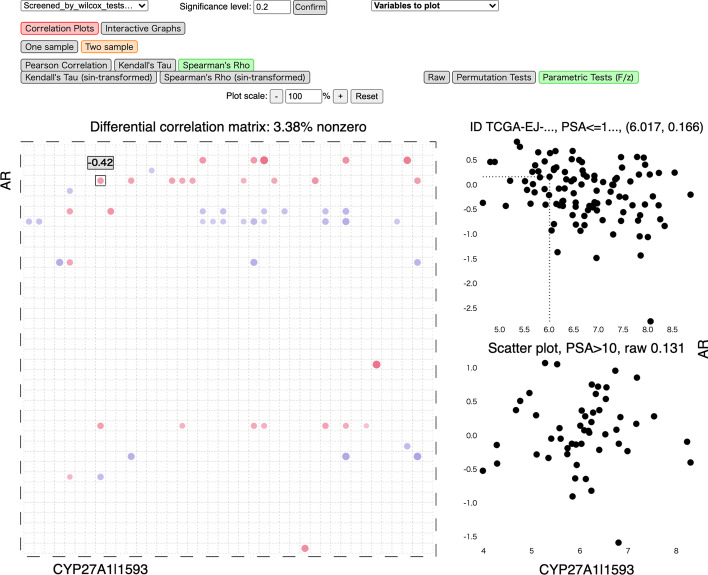
A screenshot of the interactive correlation plots using D3.js for the TCGA dataset

**Fig. 7:**
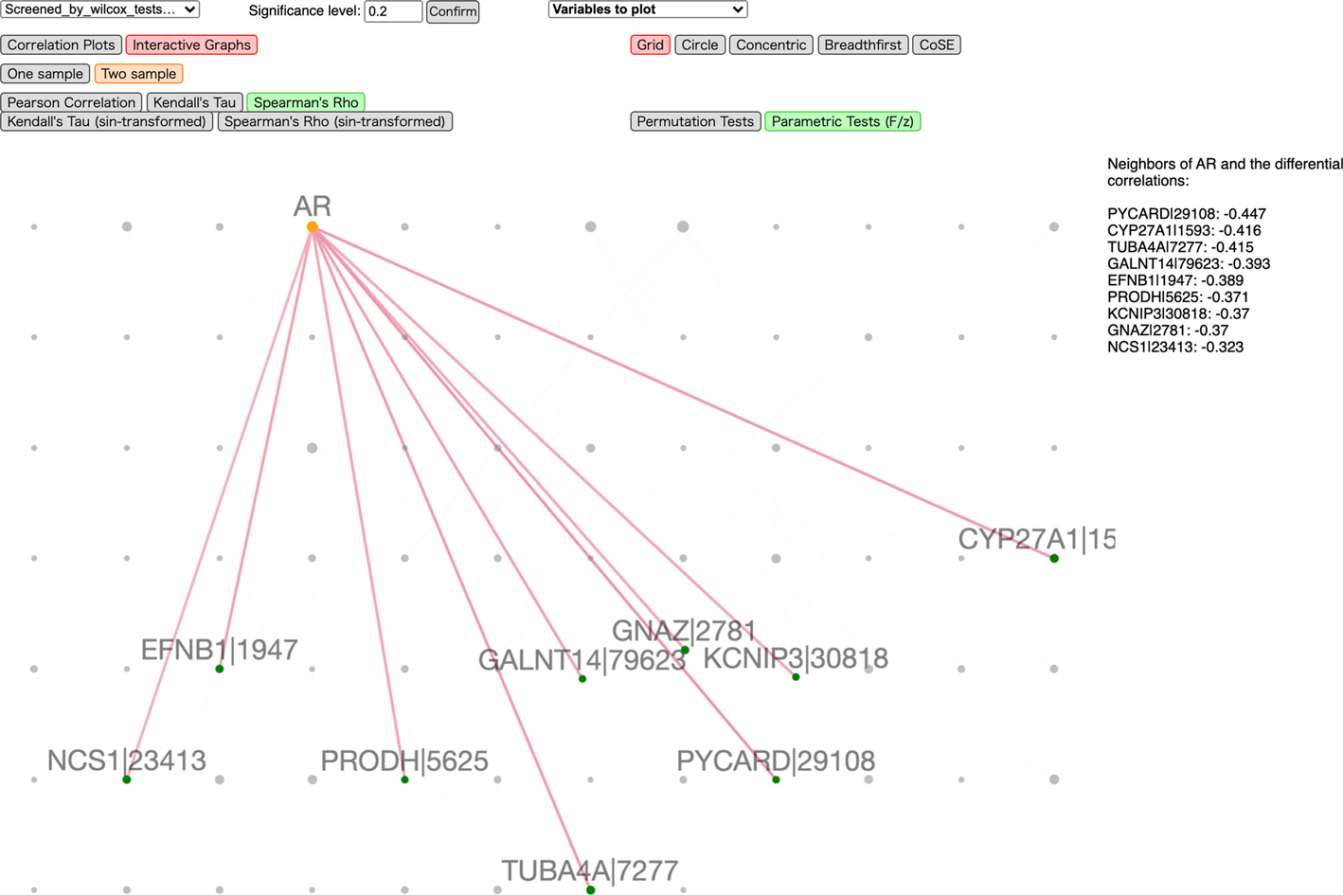
A screenshot of the interactive network plots using Cytoscape.js for the TCGA dataset

The correlation plots are shown in Fig. 6. In this two-omics case, the ${\varvec{X}}$ variables are on the vertical axis, and ${\varvec{Y}}$ are on the horizontal axis. Thus, the entry in the *j*-th row and *k*-th column now represents $\mathrm {cor}\big ({\varvec{X}}_{j}^{(t)},{\varvec{Y}}_{k}^{(t)}\big )$ for one population $t=1,2$, or their difference. Interactive network visualization for the same analysis is shown in Fig. 7. In the two-omics case, nodes in the ${\varvec{X}}$ and ${\varvec{Y}}$ groups are colored in orange and green, respectively.

### Usage

The CorDiffViz package can be used to visualize differential (cross-)correlation networks across various omics data, both for exploratory analysis as well as formal inference. Differential network analysis can be applied to, for example, gene regulatory interaction networks—to analyze the mechanistic changes resulting from responses to changed environmental conditions—or to metabolic interactions—to study the cellular processes that are differentially important [1].

The *p*-values stored in the data files represent multiple testing-adjusted *p*-values for the parametric and permutation tests. These *p*-values can be used for direct analysis, or through the visualization interface, where the user can change the default significance threshold. The interactive visualization also offers more insights into detailed changes in the networks. These features provide important insight into altered biological mechanisms, beyond what would be obtained by simply examining differential correlation heatmaps. In fact, our visualization was recently used to interrogate changes in metabolomic interaction mechanisms in Drosophila under two different diets [32], leading to new biological discoveries.

## Conclusions

We have developed an integrated R package for estimation and interactive visualization of (differential) correlation matrices/networks for two populations. The package is designed so that by calling a single R function and specifying some parameters for estimation, the estimates will be automatically saved to the local directory. Users then have access to interactive visualization by simply opening an HTML file in the browser. The package is intended to provide convenient tools for interpreting (differential) correlation networks for multi-omics data.

## Availability and requirements

**Project name**: CorDiffViz

**Project home page**: https://github.com/sqyu/CorDiffViz

**Operating system(s):** Platform independent

**Programming language**: R, HTML and Javascript

**Other requirements**: Google Chrome or Firefox

**License:** GPL-3

**Any restrictions to use by non-academics:** None.

## Abbreviations

FDR: False discovery rate
KEGG: Kyoto Encyclopedia of Genes and Genomes
PSA: Prostate-specific antigen
TCGA: The Cancer Genome Atlas
